# Comparison of Immunochromatographic Test (ICT) and Filariasis Test Strip (FTS) for Detecting Lymphatic Filariasis Antigen in American Samoa, 2016

**DOI:** 10.3390/tropicalmed6030132

**Published:** 2021-07-14

**Authors:** Meru Sheel, Colleen L. Lau, Sarah Sheridan, Saipale Fuimaono, Patricia M. Graves

**Affiliations:** 1 National Centre for Epidemiology and Health, Research School of Population Health, ANU College of Health and Medicine, The Australian National University, Acton 2601, Australia; 2 Faculty of Medicine, School of Public Health, University of Queensland, Brisbane 4006, Australia; colleen.lau@uq.edu.au; 3 Research School of Population Health, ANU College of Health and Medicine, The Australian National University, Acton 2601, Australia; 4 National Centre for Immunisation Research and Surveillance, Westmead 2145, Australia; sarah.sheridan1@health.nsw.gov.au; 5 American Samoa Department of Health, Pago Pago, AS 96799, USA; sfuima6@yahoo.com; 6 College of Public Health, Medical and Veterinary Sciences, James Cook University, Cairns 4870, Australia; patricia.graves@jcu.edu.au

**Keywords:** lymphatic filariasis, American Samoa, diagnostics, antigen

## Abstract

Circulating filarial antigen (Ag) prevalence, measured using rapid point-of-care tests, is the standard indicator used for monitoring and surveillance in the Global Program to Eliminate Lymphatic Filariasis. In 2015, the immunochromatographic test (ICT) was replaced with the filariasis test strip (FTS), which has higher reported sensitivity. Despite differences in sensitivity, no changes in recommended surveillance targets were made when the FTS was introduced. In 2016, we conducted lymphatic filariasis surveys in American Samoa using FTS, which found higher Ag prevalence than previous surveys that used ICT. To determine whether the increase was real, we assessed the concordance between FTS and ICT results by paired testing of heparinised blood from 179 individuals (63% FTS-positive). ICT had 93.8% sensitivity and 100% specificity for identifying FTS-positive persons, and sensitivity was not associated with age, gender, or presence of microfilariae. Based on these findings, if ICT had been used in the 2016 surveys, the results and interpretation would have been similar to those reported using FTS. American Samoa would have failed Transmission Assessment Survey (TAS) of Grade 1 and 2 children with either test, and community prevalence would not have been significantly different (4.1%, 95% CI, 3.3–4.9% with FTS vs. predicted 3.8%, 95%, CI: 3.1–4.6% with ICT).

## 1. Introduction

### 1.1. Lymphatic Filariasis Background and Elimination

Lymphatic filariasis (LF) is a vector-borne neglected tropical disease caused by the *Wuchereria bancrofti* and *Brugia* species of helminth worms. Globally, it is estimated that 893 million people living in 49 countries continue to be at risk of LF and require preventive chemotherapy to stop the spread of this parasitic infection [1].

The Global Programme to Eliminate Lymphatic Filariasis (GPELF), launched by the World Health Organization (WHO) in 2000, aims to interrupt LF transmission by conducting mass drug administration (MDA) in all endemic countries and providing morbidity management and disability prevention for those infected. In 2019, GPELF reported that 72 countries were LF-endemic based on progress in MDA and validation of elimination status [2]. In the WHO Western Pacific Region, the Pacific Programme to Eliminate LF (PacELF) supported 22 Pacific Island Countries and Territories. As of 2019, Cook Islands, Kiribati, Niue, the Marshall Islands, Tonga and Vanuatu have been validated by WHO for successfully achieving elimination targets [2].

### 1.2. Lymphatic Filariasis in American Samoa

In 1999, circulating filarial antigen (hereafter Ag) prevalence in American Samoa was 16.6%, measured using the Binax Now^®^ immunochromatographic test (ICT) [3]. Seven rounds of MDA with diethylcarbamazine (DEC) and albendazole were conducted between 2000 and 2006 [4], and a large household survey in 2007 showed that Ag prevalence in those aged ≥2 years had dropped to 2.3% (43/1881, 95% CI 1.66–3.07) and microfilaria (Mf) prevalence was 0.3% (5/1881, 95% CI 0.1–0.6) [5]. No further MDAs were performed until 2018.

Transmission assessment surveys (TAS) of school children in grades 1 and 2 were conducted in 2011 and 2015 using ICT and both passed WHO recommended thresholds with two and one Ag-positive children, respectively, detected in the same school both times [6]. In American Samoa, based on the likelihood that the evaluation unit passing is at least 75% if true Ag prevalence is 0.5%, and no more than 5% if the true Ag prevalence is ≥1%, the critical cut-off is six Ag-positive children [7]. Operational research studies continued between 2010 and 2016, and identified widespread Ag and antibody positivity [8,9] with higher prevalence in adults, outdoor workers, and those living in lower socioeconomic conditions, although some knowledge of filariasis was associated with lower prevalence [10].

In 2016, a school-based TAS and a population-representative household survey of LF were simultaneously conducted in American Samoa (TAS Strengthening in American Samoa). Both surveys in 2016 used the Alere^®^ Filariasis Test Strip (FTS) as the primary diagnostic tool. American Samoa failed TAS-3 after identifying nine Ag-positive children out of the 1143 tested (Ag prevalence 0.7%, 95% CI 0.3–1.8). After adjusting for sampling design, Ag prevalence in the community survey (age ≥8 years) was 6.2% (102 Ag-positive out of 2507 tested) [11]. The Ag prevalence in both surveys was notably higher than in previous surveys, suggesting possible resurgence.

### 1.3. Diagnostic Tests for LF and History of ICT/FTS

Historically, the gold standard test for diagnosis of LF was the observation of Mf on stained blood slides. This requires skilled and labour-intensive work, and slides must be made at nighttime, where LF transmission is periodic. Starting in the 1980s, immunoassays detecting circulating filarial Ag from adult worms were developed for *W. bancrofti* [12,13] using monoclonal antibodies AD12 and Og4C3, which both detect the same Ag [14]. Both tests gave quantitative readouts and needed to be carried out in specialized laboratories but could be performed on reconstituted dried blood spots [15], thus simplifying shipping of blood samples from endemic areas if local laboratory expertise was not available.

The ELISA test developed by Weil et al. [12] using AD12 was subsequently converted to a rapid test format, the ICT [16], that came into widespread use at the start of the GPELF. The ICT test uses 100 μL of whole blood taken directly from a finger-prick or venous sample or collected into tubes with anticoagulants (heparin or EDTA). Studies comparing the accuracy of the ICT to other Ag ELISAs or DNA-detection methods on blood and urine concluded that the ICT test was the most useful for programmatic field surveys [17], despite the lack of a quantitative readout from a lateral flow test.

To reduce the cost and amount of blood needed to perform a rapid point-of-care test as well as improve the shelf life and storage conditions, the FTS was developed using the same Ag as the ICT but in a new format [18]. Currently, non-anticoagulated blood is recommended for FTS, although many studies used blood collected in heparinised microtainers and tested within 24 h. Comparisons between ICT and FTS in controlled laboratory conditions using 227 archived serum or plasma samples showed that the two tests had similarly high rates of sensitivity and specificity and >99% agreement [18]. However, in a field study in Liberia, the FTS produced 26.5% more positive results compared to ICT (124/503 versus 98/503) [18].

In 2014, a WHO technical meeting reviewed 15 programmatic surveys comparing FTS with ICT and found that in 10 of these surveys, there were more FTS-positive than ICT-positive cases. The surveys were conducted in areas that differed in Ag prevalence, MDA history, and type of blood sample used [19], with studies conducted in post-MDA areas tending to show lower concordance between the two tests. Nevertheless, the Neglected Tropical Diseases Strategic and Technical Advisory Group’s Monitoring and Evaluation Sub-group on Disease Specific Indicators were satisfied with the diagnostic characteristics of the FTS compared with ICT [19]. There was an agreement that increased sensitivity of the FTS above that of the ICT was acceptable to the GPELF, and that guidance on implementing a TAS and critical cut-off numbers would not be changed. Subsequent published field studies found that FTS detected more Ag-positive persons compared with ICT in Indonesia (6.4% vs. 5.3%) and Sri Lanka (5.0% vs. 2.1%), with ratios of positive FTS/ICT of 1.22 in Indonesia and 2.33 in Sri Lanka. In both countries, the Ag detection rate measured by FTS versus ICT was similar in children aged <11 years and in females but not in males, who had prevalence two to four times higher with FTS than ICT [20]; these differences may be related to different Ag levels. In Congo and Cote d’Ivoire, paired tests conducted on 3682 individuals found that Ag prevalence were 8% and 22% higher by FTS than by ICT in pre-MDA and post-MDA settings, respectively [21].

Given the lower concordance shown between FTS and ICT in post-MDA settings, there was initial concern that the findings of higher Ag prevalence in 2016 in American Samoa [11] using FTS may not be real because previous surveys utilised ICT, Og4C3 ELISA [8], or both [9,10]. Therefore, it was prudent to assess concordance of the results between the two tests in American Samoa, particularly considering experiences being reported by other countries. For this purpose, we used both ICT and FTS on the same blood samples from a subset of participants in the 2016 surveys in American Samoa to examine the concordance of results between the two tests.

## 2. Materials and Methods

American Samoa is a US Territory in the South Pacific (14.2710° South, 170.1322° West), consisting of small, inhabited islands with a total population of ~55,519 persons living in ~70 villages. Over 90% of the population resides on the main island of Tutuila and the adjacent island of Aunu’u [22]. The remote Manu’a islands were not included in this study, as seroprevalence studies on samples collected in 2010 did not identify any Ag-positive persons [8]. The current study was conducted in 2016 on Tutuila and Aunu’u.

### 2.1. Specimen Collection and Testing

We collected 200 μL of finger prick blood sample into heparinised microtainers or ~8 mL of venous blood samples into tubes with heparin anticoagulant. Samples collected in the community were kept cool and transported to the American Samoa Community College or the Department of Health Public Health Laboratory on the same day and tested using FTS and ICT on the same or following day in a controlled laboratory environment [11].

Samples were tested using ICT and FTS according to the manufacturer’s directions. Briefly, either 100 µL (ICT) or 75 µL (FTS) of blood were placed in the sample pad and left to migrate for 10 min. At the end of the 10 min, results were recorded as positive, negative, or invalid. For participants who were FTS-positive, we prepared slides for microscopic examination of Mf, as described previously [7].

### 2.2. Selection of Samples for Comparison of ICT and FTS

This study was conducted during the 2016 TAS Strengthening study in American Samoa [11]. The following samples from the 2016 surveys were selected for paired comparisons of ICT and FTS [11]:

1. Follow-up visits (at home) of Ag-positive children identified in the school-based TAS, and their family members.
2. Follow-up visits (in clinic) of Ag-positive participants and their family members from the community survey of randomly selected households.
3. Additional convenience sample of residents in Fagali’i, a high-prevalence village. Due to the remote location of Fagali’i, some participants were tested by FTS in the village so that treatment could be provided immediately to Ag-positive persons. Remaining samples were tested in a controlled laboratory environment.

As only 200 ICT cards were available, we applied a pragmatic approach to selecting samples for testing. We selectively sampled from the above groups where we could identify sufficient numbers of Ag-positive and -negative persons for comparing the two diagnostic tests.

### 2.3. Data Collection and Analyses

For all study participants, we collected demographic data using electronic questionnaires administered by bilingual field research assistants in Samoan or English according to each participant’s preference, as described previously [11].

The outcome measures were positive FTS or ICT tests. We undertook descriptive analyses for age and gender, and compared simple proportions using McNemar’s chi-squared test. In the absence of a gold standard diagnostic test for the detection of LF Ag, we used FTS as a reference standard for comparison with ICT [23]. Kappa agreement statistic was used to analyse the concordance between ICT against FTS as a reference standard. We calculated the sensitivity and specificity of ICT against FTS using the *diagt* command in Stata 15. *p* Values of <0.05 were considered statistically significant. We used the binomial exact method to estimate 95% confidence intervals (CI).

### 2.4. Informed Consent, Ethics Approvals, and Cultural Considerations

For the school-based survey, permissions were sought from school principals along with signed consent forms from parents/guardians, and assent was sought from all participants. For the community survey, we obtained signed informed consent from adult participants or from parents/guardians of those aged <18 years accompanied by verbal assent. Ethics approvals for the study were granted by the American Samoa Institutional Review Board and the Human Research Ethics Committee at the Australian National University (protocol number 2016/482). The study was conducted in collaboration with the American Samoa Department of Health and the American Samoa Community College. Permissions were also granted by the Department of Education and the Department of Samoan Affairs, respectively. Surveys were implemented in a culturally appropriate and sensitive manner, as previously described [11].

All FTS-positive persons (excluding pregnant women who were advised to seek treatment after delivery) were provided treatment with DEC and albendazole according to national guidelines.

## 3. Results

A total of 179 individuals were tested using FTS and ICT on the same blood sample. Of these, seven (3.9%) were Ag-positive school children, six (3.4%) were household members of Ag-positive school children, and 166 (92.7%) were community members including those from randomly selected households for the community survey and the additional convenience sample of residents from Fagali’i (Table 1). FTS identified more Ag-positive (112/179, 62.6%, 95% CI: 55.0–69.7%) persons compared with ICT (105/179, 58.7%, 95% CI: 51.1–65.9%) but the difference in estimated overall prevalence was not statistically significant (*p* > 0.05). Slides were available for 166 of the FTS-positive participants, and 28 (15.6%) were Mf-positive. Of the 179 samples tested, 105 (58.7%) were Ag-positive by both FTS and ICT, 67 (37.4%) were Ag-negative with both tests, seven (3.9%) were FTS-positive but ICT-negative, and zero were FTS-negative but ICT-positive (Table 1). We observed 96.1% agreement with a kappa value of 0.92. Using FTS as the reference, we assessed the accuracy of ICT in detecting Ag-positive persons and found that ICT was 93.8% (95% CI: 87.5–97.5%) sensitive and 100% (95% CI: 94.6–100%) specific. Within subgroups by age (≤14 years, 15–44 years, and ≥45 years) or gender, there were no differences in the sensitivity and specificity of ICT (Table 2). Of the seven discordant results (ICT-negative/FTS-positive), all were Mf-negative (one female aged 9 years, two males 15–44 years, and four males ≥45 years). For those who were FTS-positive, we compared ICT sensitivity and specificity between Mf-positive (n = 28) and Mf-negative (n = 72) persons and did not find any statistically significant difference with this sample size (Table 2).

Applying 93.8% sensitivity and 100% specificity for ICT to the 2016 school-based TAS-3, where nine Ag-positive children were identified using FTS, we would expect to have identified eight Ag-positive children using ICT, which is still above the critical cut-off of six Ag-positive children for failing TAS in these surveys. In contrast, if the 2011 TAS-1 and the 2015 TAS-2 had used FTS for detecting LF antigen, we would have identified the same number of Ag-positive persons (two and one, respectively) as were found when using ICT. Therefore, the pass/fail result of each TAS survey in American Samoa would have been the same with either test. If we apply the same assumptions about test characteristics to the 2016 community survey, we would have found similar crude Ag prevalence of 3.8% (CI 3.1–4.6%) using ICT compared with 4.1% (CI 3.3–4.9%) using FTS.

## 4. Discussion

In this 2016 study, we present findings comparing FTS and ICT in American Samoa in a post-MDA setting. The results show that ICT had lower sensitivity (93.8%) than but the same specificity (100%) as FTS. The difference in Ag prevalence detected by the two tests was not statistically significant. There was a high level of overall agreement between FTS and ICT, and results of the 2016 survey would have been very similar with either test. Importantly, none of the ICT-positive cases were FTS-negative, confirming that there were no diagnostic losses when FTS was used [19]. Therefore, resurgence of transmission, and not differences in diagnostic test characteristics, was the most likely reason for higher Ag prevalence in American Samoa in 2016 compared with previous studies [5,8,9].

Other studies have suggested that ICT may be less sensitive than FTS in males [20], but we did not observe a significant difference by sex. American Samoa is a post-MDA setting (even though this survey was conducted ten years after the last MDA); however, we did not observe lower sensitivity for ICT of the magnitude seen in other post-MDA settings [21].

Although the 2007 survey in American Samoa relied solely on ICT for Ag detection [5], other studies on Ag prevalence conducted between 2010 and 2014 in American Samoa have used ICT, Og4C3 ELISA, or a combination of both [8,9]. Based on the findings of Gass et al. [17], Masson et al. [15], and Gounoue-Kamcuso et al. 2015 [24], ELISAs such as Og4C3 are more accurate than ICT, especially in areas of low prevalence. Nevertheless, rapid tests were chosen as the main test for LF monitoring and evaluation purposes worldwide due to convenience and adequate test performance [7].

Considering the test characteristics of FTS, lower cost (USD < 1.50 versus USD~3.0), greater temperature stability, and reduced blood volumes required, our study also provides further evidence in support of the use of FTS as the primary diagnostic test for LF under the GPELF.

## Figures and Tables

**Table 1:** Comparison of Filariasis Test Strip (FTS) and immunochromatographic test (ICT) for detecting LF antigen-positive persons, American Samoa, 2016.

	FTS-Positive N (%)	FTS-Negative N (%)	Total N (%)
**ICT-positive**	105 (93.8)	0 (0)	105 (58.7)
**ICT-negative**	7 (6.2)	67 (100.0)	74 (41.3)
**Total**	112 (100.0)	67 (100.0)	179 (100.0)

**Table 2:** Demographic details, sensitivity, and specificity for study participants (N = 179) tested for LF antigen with both Filariasis Test Strip (FTS) and immunochromatographic test (ICT), American Scheme 2016.

	Number Tested	FTS-Positive (%)	ICT-Positive (%)	Sensitivity of ICT Compared to FTS (%)	Specificity of ICT Compared to FTS (%)
Overall	179	112 (62.6)	105 (58.7)	93.8 (87.5–97.5)	100 (94.6–100)
**Age groups**					
≤14 years	37	20 (54.1)	19 (51.4)	95.0 (75.1–99.9)	100 (80.5–100.0)
15–44 years	74	42 (56.8)	40 (54.1)	95.2 (83.8–99.4)	100 (89.1–100.0)
≥45 years	68	50 (73.5)	46 (67.7)	92.0 (80.8–97.8)	100 (81.5–100.0)
Sex					
Male	107	76 (71.0)	70 (65.4)	92.1 (83.6–97.0)	100 (88.8–100.0)
Female	72	36 (50.0)	35 (48.6)	97.2 (85.5–99.9)	100 (90.3–100.0)
**FTS-positive/Mf status**					
Mf-positive	28 ^#^	28 (100.0)	28 (100.0)	100 (87.7–100.0) *	100 ^$^
Mf-negative	72 ^#^	72 (100.0)	65 (90.3)	90.3 (81.0–96.0)	100.0 (94.6–100.0)

* one-sided, 97.5% confidence interval ^#^ slides were only available for persons who were FTS-positive. ^$^ FTS and ICT results were 100% concordant for persons who were FTS- and Mf-positive.

## Data Availability

We are unable to provide individual-level antigen prevalence data and demographic data because of the potential for breaching participant confidentiality. The communities in American Samoa are very small, and individual-level data such as age, sex, and village of residence could potentially be used to identify specific persons. For researchers who meet the criteria for access to confidential data, the data are available on request from the Human Ethics Officer at the Australian National University Human Research Ethics Committee, email: human.ethics.officer@anu.edu.au.
